# Extensive localization of long noncoding RNAs to the cytosol and mono- and polyribosomal complexes

**DOI:** 10.1186/gb-2014-15-1-r6

**Published:** 2014-01-07

**Authors:** Sebastiaan van Heesch, Maarten van Iterson, Jetse Jacobi, Sander Boymans, Paul B Essers, Ewart de Bruijn, Wensi Hao, Alyson W MacInnes, Edwin Cuppen, Marieke Simonis

**Affiliations:** 1 Genome Biology Group, Hubrecht Institute-KNAW and University Medical Center Utrecht, Uppsalalaan 8, 3584, CT Utrecht, The Netherlands; 2 Ribosome Biogenesis and Disease Group, Hubrecht Institute-KNAW and University Medical Center Utrecht, Uppsalalaan 8, 3584, CT Utrecht, The Netherlands; 3 Department of Medical Genetics, University Medical Center Utrecht, 3584, CG Utrecht, The Netherlands

## Abstract

**Background:**

Long noncoding RNAs (lncRNAs) form an abundant class of transcripts, but the function of the majority of them remains elusive. While it has been shown that some lncRNAs are bound by ribosomes, it has also been convincingly demonstrated that these transcripts do not code for proteins. To obtain a comprehensive understanding of the extent to which lncRNAs bind ribosomes, we performed systematic RNA sequencing on ribosome-associated RNA pools obtained through ribosomal fractionation and compared the RNA content with nuclear and (non-ribosome bound) cytosolic RNA pools.

**Results:**

The RNA composition of the subcellular fractions differs significantly from each other, but lncRNAs are found in all locations. A subset of specific lncRNAs is enriched in the nucleus but surprisingly the majority is enriched in the cytosol and in ribosomal fractions. The ribosomal enriched lncRNAs include *H19* and *TUG1*.

**Conclusions:**

Most studies on lncRNAs have focused on the regulatory function of these transcripts in the nucleus. We demonstrate that only a minority of all lncRNAs are nuclear enriched. Our findings suggest that many lncRNAs may have a function in cytoplasmic processes, and in particular in ribosome complexes.

## Background

The importance of noncoding RNA transcripts for key cellular functions has been well established by studies on for example *XIST*[1], which acts in X-chromosome silencing, and *TERC*[2], which functions in telomeric maintenance. Genomic studies performed in the last decade have shown that these are likely not isolated examples as many more long non protein-coding transcripts were identified [3-5]. Although it remains to be demonstrated that all of these transcripts have specific functions [6], functional studies showing the importance of long noncoding RNAs (lncRNAs) as regulators in cellular pathways are accumulating rapidly (for example, [7-12]). However, the function and the mechanisms of action of the majority of lncRNAs are still unexplored [13].

Cellular location is an important determinant in understanding the functional roles of lncRNAs. Subcellular RNA sequencing (RNA-seq) has been performed to explore the differences between nuclear, chromatin-associated and cytoplasmic transcript content in several cell lines [14] and macrophages [15]. Derrien *et al*. [3] specifically estimated the relative abundance of lncRNAs in the nucleus versus the cytosol and concluded that 17% of the tested lncRNAs were enriched in the nucleus and 4% in the cytoplasm. This is in line with the function of some individual lncRNAs, such as *NEAT1* and *MALAT1*, which were shown to be involved in nuclear structure formation and gene expression regulation [7,8]. However, it has been argued that relative enrichment does not mean that the absolute number of transcripts for each lncRNA is also higher in the nucleus [13]. Some lncRNAs were enriched in the cytoplasm and ribosome profiling demonstrated that part of the cytoplasmic lncRNAs is bound by ribosomes [16]. More detailed characterization of the ribosome profiling data showed that ribosomal occupation of lncRNAs does not match with specific marks of translation [17].

While these results suggest diverse roles of lncRNAs in different cellular compartments and biological processes, comprehensive knowledge on the relative abundances of lncRNAs in ribosomes, the cytosol and the nucleus is currently still lacking. Moreover, as ribosomal profiling measures single sites in RNA molecules that are occupied by ribosomes, this technique does not yield information on the number of ribosomes that are present per single (physical) lncRNA transcript [18]. In a different method, named ribosomal fractionation, a cytosolic size separation is performed that results in the isolation of translation complexes based on the amount of ribosomes associated per transcript [19]. This method has been used in combination with microarrays to analyze ribosomal density on protein-coding transcripts [20-22] but not on lncRNAs.

Here we perform subcellular RNA-seq on nuclei, cytosol and mono- and polyribosomes separated by ribosomal fractionation. Our data show relative enrichment of specific lncRNAs in the nucleus, but also demonstrate that most lncRNAs are strongly enriched in the cytosol and in ribosomal fractions.

## Results

### Nuclear, cytosolic and ribosomal fractions differ in transcript content

**Figure 1:**
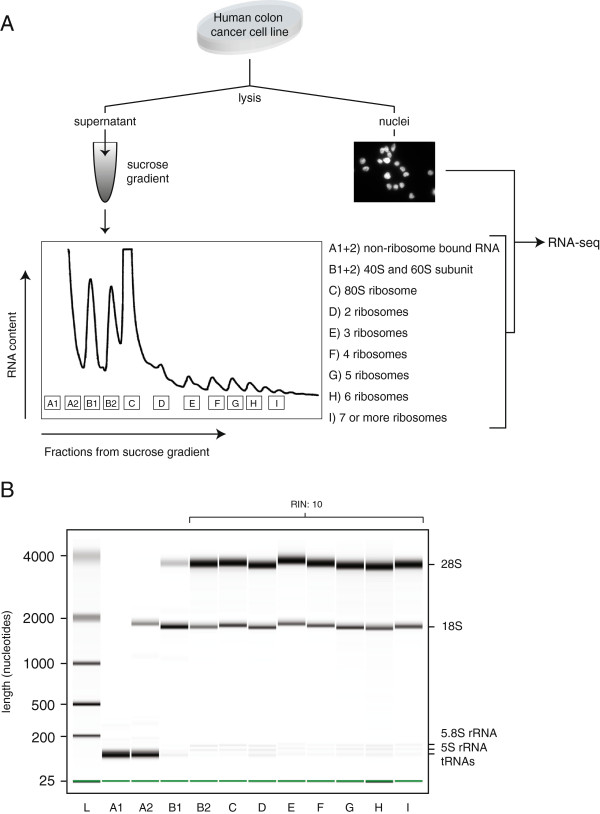
**Experimental workflow and quality control. (A)** Cells were lysed and the complete cytosolic fraction was used for ribosomal fractionation. Pelleted nuclei and nine fractions (indicated A to I) derived from the ribosomal fractionation were subsequently used for RNA isolation and strand-specific RNA-seq. Fractions A1 and A2 as well as B1 and B2 were merged prior to the RNA-seq. **(B)** 2100 Bioanalyzer RNA 6000 Pico results showing the integrity of the collected RNA samples obtained by ribosomal fractionation. Each ribosomal fraction has an RNA integrity value of 10. These results also show the sample-specific content of tRNAs, 5S, 5.8S, 18S and 28S rRNA, which nicely indicate the purity of the fractionation. RIN, RNA integrity.

Different subcellular RNA fractions were isolated from the human cell line LS-174 T-pTER-*β*-catenin [23] (Figure 1). The cells were first subjected to a mild lysis after which the nuclei were separated from the cytosol and other organelles by centrifugation. Microscopic inspection and nuclear staining confirmed the presence of clean nuclei in the pellet and thus the co-sedimentation of the rough endoplasmic reticulum-derived ribosomes with the cytosolic supernatant (Additional file 1). The cytosolic sample was fractionated further using a sucrose gradient and ultracentrifugation, which sediments the sample components based on size and molecular weight. UV was used to measure the RNA content of the fractions and the amount of ribosomes in each of the fractions was established based on the resulting distinct peak pattern. We isolated each of the fractions containing one, two, three, four, five and six ribosomes and the fraction containing seven or more ribosomes. In addition, we isolated the fraction that contained the cytosolic part without ribosomes, which we will refer to as the ‘free cytosolic’ sample. RNA molecules in the free cytosolic fraction are, however, associated with various other types of smaller protein complexes that reside in the cytosol. The fractions containing 40S and 60S ribosomal subunits were also extracted and these two samples were pooled for further analysis. The RNA of three ribosomal fractionation experiments was pooled to level out single experimental outliers. Through this experimental setup we obtained a complete set of subcellular samples from which RNA was extracted.

Strand-specific RNA-seq was performed after rRNA depletion on all the subcellular samples and for each we obtained at least six million aligned reads. The GENCODE annotation [24] of coding and noncoding transcripts was used to establish the read counts per gene (Additional file 2). In our data analyses, we considered three types of transcripts: protein-coding transcripts; small noncoding RNAs (sncRNAs), which included small nuclear RNAs (snRNAs) and small nucleolar RNAs (snoRNAs); and lncRNAs, which included antisense transcripts, long intergenic noncoding RNAs and processed transcripts (these were transcripts that did not contain an open reading frame (ORF) and could not be placed in any of the other categories) [3]. We left out some small RNAs such as miRNAs, because these were not captured in our experimental setup. Also, to prevent false assignments of sequencing reads to noncoding transcripts, we did not consider lncRNAs in which the annotation partially overlapped with protein-coding transcripts on the same strand. We selected expressed transcripts using a stringent threshold to allow us to reliably detect quantitative differences. Our expressed transcript set contained 7,734 genes including 7,206 protein-coding genes, 152 lncRNAs (46 antisense transcripts, 71 long intergenic noncoding transcripts and 35 processed transcripts) and 376 sncRNAs (134 snoRNAs and 242 snRNAs).

**Figure 2:**
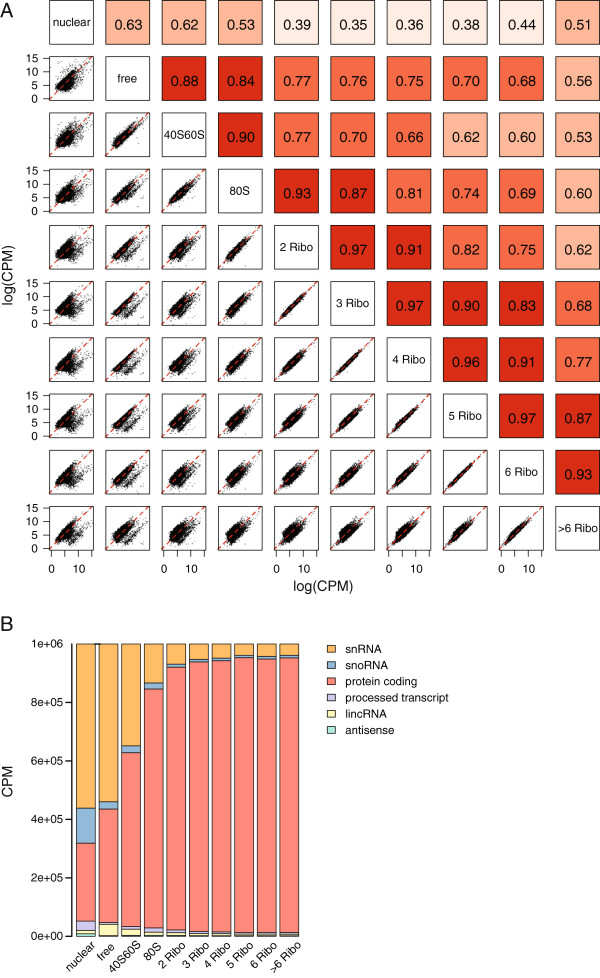
**Subcellular RNA fractions have a different transcript composition. (A)** Scatter plot and correlation matrix of all sequenced samples. The color intensity of the correlation boxes (r values) depicts the relative strength of the correlation, ranging between 0.39 and 0.97. **(B)** RNA species content of each sequenced fraction in counts per million. CPM, counts per million; lincRNA, long intergenic noncoding RNA; snoRNA, small nucleolar RNA; snRNA, small nuclear RNA.

To determine the similarity of the RNA content of the different subcellular samples we analyzed the correlations between each sample pair (Figure 2A). The highest correlations were seen between ribosomal fractions, ranging from 0.60 to 0.97. By contrast, the correlations between the different ribosomal fractions and the nuclear sample ranged from 0.35 to 0.53. We investigated the source of the variable correlation between subcellular RNA samples by comparing the origin of the RNA reads from each fraction (Figure 2B). This analysis showed that more than half of the reads in the nuclear sample aligned to sncRNAs and this group of small RNAs was visible as a distinct cloud in the comparative scatter plots (Figure 2A and Additional file 3). The ribosomal fractions primarily consisted of protein-coding genes as expected, but highly expressed lncRNAs were also clearly present. Because these read count distributions did not directly translate into transcript composition of the different samples, we also analyzed the sample composition based on reads per kilobase per million. This resulted in essentially the same distribution among the samples, but the relative contribution of sncRNAs was larger (Additional file 4).

Combined, these analyses show that subcellular RNA samples have very different compositions and that lncRNAs are found in each of the subcellular RNA samples.

### Long noncoding RNAs are primarily enriched in the cytosol and in the ribosomal fractions

**Figure 3:**
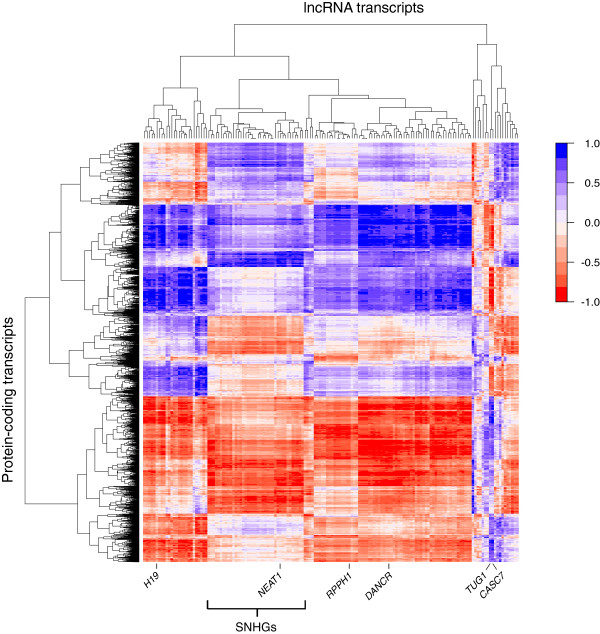
**Long noncoding RNAs show a subcellular distribution similar to specific groups of protein-coding transcripts.** Heatmap of the Spearman-Rank correlation between the each of the 152 expressed lncRNAs and 7,206 expressed protein-coding transcripts across the subcellular RNA samples. Strong correlations are shown in blue, anti-correlations are shown in red. Six frequently studied lncRNAs with varying correlations to protein-coding transcripts are highlighted at the bottom together with a large cluster that harbors the majority of expressed snoRNA host genes. lncRNA, long noncoding RNA.

The clear difference in composition of the subcellular RNA samples raises the question how individual transcripts are distributed among the samples and in particular how lncRNAs behave compared to protein-coding transcripts. Therefore we investigated the distribution of each lncRNA across the cellular fractions versus the distribution of each protein-coding transcript (Figure 3). The correlation between each protein-coding transcript-lncRNA pair was calculated and the obtained scores depicted in a clustered heatmap (Figure 3). A high correlation between two transcripts in this heatmap meant that the two showed a very similar distribution across all different subcellular samples. This analysis showed that there are several different groups of lncRNAs that can be distinguished based on their correlation with protein-coding transcripts. Each group of lncRNAs had specific sets of positively correlated and negatively correlated protein-coding transcripts. Examples of such groups are the noncoding snoRNA host genes, that all showed very similar correlation profiles (Figure 3). A few lncRNAs, including *TUG1* and *CASC7*, had a more specific correlation profile. These results show that there is no general negative correlation between cellular localization of lncRNAs and protein-coding transcripts, but that the relationships are complex.

**Figure 4:**
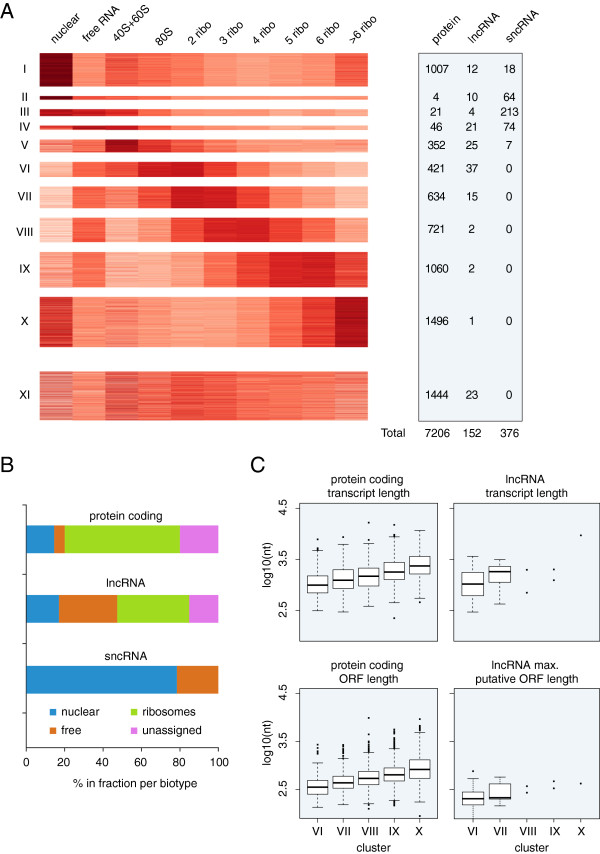
**RNA species show specific distributions across the subcellular RNA samples. (A)** Heatmap display of the 11 clusters and the number of protein-coding, lncRNA and sncRNA transcripts present in each cluster. **(B)** Summarizing plot showing the distribution of the three types of transcripts over the four major types of clusters that could be derived from the analysis in **(A)**. **(C)** Boxplots of the total transcript length and the maximum (potential) open reading frame of protein-coding transcripts and lncRNAs in clusters VI to X. lncRNA, long noncoding RNA; ORF, open reading frame; sncRNA, short noncoding RNA.

To reduce this complexity and to focus on the distribution of protein-coding transcripts and non-protein-coding RNAs across the subcellular fractions we applied model-based clustering on the normalized read counts per transcript [25]. We applied the clustering algorithm using variable amounts of clusters and found that a separation in 11 clusters best describes the data (Figure 4A and Additional files 5 and 6). All RNA-seq transcript levels were normalized to the total amount of sequencing reads produced per sample. Therefore, the normalized value of a transcript depended on the complexity of the sample (number of different transcripts) and the expression level of all other transcripts. Because of the large fraction of reads that arose from sncRNAs, we tested the effect of omitting these RNAs from the dataset and found that this did not affect the clustering results (Additional file 7). The final set of 11 clusters included one cluster (XI) containing transcripts that did not show an obvious enrichment in any of the samples, and 10 clusters (I to X) containing genes that did show a specific cellular localization. Clusters I, II and III all contained transcripts enriched in the nucleus and depleted from the ribosomal fractions, but the clusters differed from each other based on the relative transcript levels in the free cytosolic and the 40S/60S sample. Cluster IV and V contained transcripts enriched in the free cytosolic sample and transcripts enriched in the 40S/60S sample, respectively. Clusters VI through X contained transcripts enriched in specific ribosomal fractions. Each of these ribosomal-enriched clusters also showed mild enrichment in the free cytosolic sample, except for cluster X, which was higher in the nucleus than in the free cytosol.

Overall, we consider clusters I, II and III as enriched in the nucleus; IV and V as enriched in the ribosome-free cytosol; and VI, VII, VIII, IX and X as enriched in the ribosomes. The distribution of protein-coding genes and sncRNAs among the clusters was largely as expected (Figure 4B). Protein-coding transcripts were present in all of the clusters, but the majority (60%) was found in the ribosomal-enriched clusters. Nonetheless, 14% of the protein-coding transcripts were found in the nuclear clusters and depleted from ribosomes, suggesting that this large part of the protein-coding transcripts is not actively translated or has a rapid turn-over rate in the cytosol. sncRNAs were found only in the nuclear and ribosome-free cytosolic clusters and not in the ribosomal clusters, which matched expectations and thus demonstrated the effectiveness of the fractionation. The majority of the sncRNAs could be found in cluster III, showing high levels both in the nucleus and free in the cytosol, suggesting that many of these small RNAs shuttle between nucleus and cytoplasm.

The most notable result was the distribution of the lncRNAs among the different clusters. In line with previous analyses [3], 17% of the lncRNAs were found in one of the nuclear clusters (Figure 4B). However, in contrast to previous studies, a relatively large part of the lncRNAs (30%) was located in clusters enriched in the ribosome-free cytosol and a striking 38% was present in ribosome-enriched clusters. As noted above, the transcript levels determined by RNA-seq represent which part of the total RNA samples can be assigned to each specific transcript. These results thus show that many individual lncRNAs (38% of the expressed lncRNAs) make up a larger part of specific ribosomal fractions than of the nuclear sample.

Although the correlations between ribosomal fractions were high (Figure 2A), these clustering results highlight the transcripts that are differential across the ribosomal samples. Previous studies have shown that many protein-coding transcripts are not evenly distributed among the ribosomal fractions, but rather show enrichment for a specific number of ribosomes [20,21]. The coding sequence length was shown to be a major determinant of the modular amount of ribosomes per transcript. In our data, the total transcript length of protein-coding transcripts in the five ribosomal clusters also increased with increasing numbers of ribosomes present (Figure 4C). For lncRNAs, we could determine such a relationship only between cluster VI (80S and two ribosomes) and VII (three and four ribosomes), because the number of lncRNAs in the clusters with a higher number of ribosomes was too low (Figure 4A). lncRNAs in cluster VII (three and four ribosomes) had a longer transcript length, longer maximum putative ORF length and more start codons than the lncRNAs in cluster VI (80S and two ribosomes) (Figure 4C and Additional file 8). However, the maximum ORF lengths of the lncRNAs were much shorter than the coding sequence length of the protein-coding genes in the same cluster, so these ORF lengths likely do not determine the number of ribosomes associated with a lncRNA.

Combined, these analyses showed that many lncRNAs were enriched in specific subcellular fractions. Although some lncRNAs were enriched in the nucleus, many more were enriched in the cytosolic and ribosomal fractions.

### Known long noncoding RNAs are enriched in different ribosomal fractions

**Figure 5:**
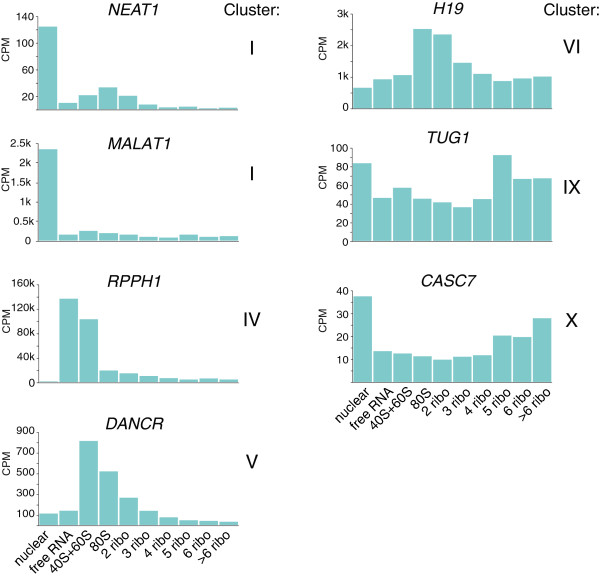
**Individual long noncoding RNAs are differentially distributed across subcellular samples.** The normalized read counts of seven lncRNAs that are found in different clusters in Figure 4. CPM, counts per million.

The cellular localization of some lncRNAs was established previously and our results were largely in agreement with earlier findings. For example, *MALAT1* and *NEAT1*, which are known to regulate nuclear processes such as gene expression [8] and the formation and maintenance of nuclear speckles and paraspeckles [7,26] respectively, were located in nuclear cluster I (Figure 5). Another lncRNA with a known nuclear function is *TUG1* (Figure 5), which is involved in the upregulation of growth-control genes [27]. We indeed found high levels of *TUG1* in the nucleus, but the transcript also showed a clear enrichment in the fractions containing five or six ribosomes. The association of *TUG1* with polysomes has not been described previously and suggests mechanisms of action in regulation of translation at the ribosome in addition to the previously described function in the nucleus.

In the ribosome-free cytosolic sample we found enrichment of lncRNAs that are known components of cytosolic complexes, for example *RPPH1* and *RN7SL1. RPPH1* is part of ribonuclease P [28] and *RN7SL1* is part of the signal recognition particle that mediates co-translational insertion of secretory proteins into the lumen of the endoplasmic reticulum [29,30]. In addition, we also found many unstudied lncRNAs in the free cytosolic fraction. In cluster V, which showed enrichment in the 40S/60S sample, we found the lncRNA *DANCR* (Figure 5). *DANCR* was recently shown to be involved in retaining an undifferentiated progenitor state in somatic tissue cells [10] and osteoblast differentiation [31]. The exact mechanisms through which *DANCR* acts are unknown, but our data suggest a role for *DANCR* predominantly outside of the nucleus. One of the most abundant lncRNAs in our data was the evolutionary conserved and imprinted *H19*. This transcript is a strong regulator of cellular growth and overexpression of *H19* contributes to tumor initiation as well as progression, making it a frequently studied noncoding RNA in cancer [9,32]. An enrichment of *H19* in the cytoplasm over the nucleus has previously been observed [3]. Here, we found only moderate levels of *H19* RNA in the nucleus and ribosome-free cytosol, but very high levels of *H19* RNA associated with ribosomes (Figure 5). This predominant association with ribosomes suggests a possible role for *H19* in the regulation of the translation machinery and, more specifically, in polysomal complexes.

*CASC7* was the only lncRNA that was enriched in the sample with seven or more ribosomes. Even though *CASC7* has been identified as a cancer susceptibility candidate, not much is known about this transcript. Our data indicate that it is sequestered to large polysomal complexes and it may thus function in regulation of translation.

Using quantitative PCR, we confirmed the enrichment of *NEAT1* and *MALAT1* in the nucleus and the enrichment of *TUG1* and *H19* in ribosomes (Additional file 9).

These results reveal the subcellular enrichment of known and unknown lncRNAs and suggest that many lncRNAs function primarily outside the nucleus.

## Discussion

We performed transcriptome analyses on subcellular samples of the human cell line LS-174 T-pTER-*β*-catenin and found that the lncRNAs that were expressed in these cells were present in all subcellular fractions, but the majority of the expressed lncRNAs were enriched in the cytosol and in ribosomes. Our data partially contradict an earlier study in which most lncRNAs were found enriched in the nucleus, compared to the cytoplasm [3]. This discrepancy could have resulted from the use of different cell types, but may also have partially resulted from measuring and comparing relative enrichments between multiple samples. Measuring the whole cytoplasm would thus result in different enrichment values compared to analysis of a specific subset of the cytoplasm, such as the ribosomes.

We are not the first to find lncRNAs associated with ribosomes. Ribosome profiling in mouse embryonic stem cells also showed examples of these interactions and our results overlap with the results from that study [16]. For example, both our work and work from Ingolia *et al*. pinpoint the lncRNA *NEAT1* as not highly associated with ribosomes. The results for *MALAT1* are more intricate, as we found that *MALAT1* was strongly enriched in the nucleus, but but previous work showed binding of ribosomes to the 5-part of this lncRNA [16,33]. It is possible that a small proportion of the *MALAT1* transcripts is bound by ribosomes. It is also likely that ribosomal association with lncRNAs is specific to cell type, growth condition and organism.

Our data add significant insight into ribosomal association of lncRNAs, because ribosomal profiling and ribosomal fractionation provide different, yet complementary, information. In ribosome profiling, specific binding sites of ribosomes are measured and the amount of binding is estimated based on the total amount of reads in the ribosome-bound versus the total RNA sample. By applying ribosomal fractionation we can directly measure the amount of ribosomes associated per lncRNA. Moreover, we measured the full range of subcellular samples including free cytosolic and nuclear RNA in one analysis. From our data we can conclude that many lncRNAs are found in complexes that contain multiple ribosomes. In addition, the enrichment of lncRNAs in ribosomal fractions shows that many lncRNAs make up a relatively larger part of the ribosomal samples than of the nuclear sample. This did not change when sncRNAs were excluded from the analyses. It should be noted that the identification of the ribosomes was based on size fractionation and RNA content. We can therefore not fully exclude that the lncRNAs are associating with protein complexes of sizes similar to the specific amounts of ribosomes [34]. However, these thus far unknown complexes would have to be present in such high quantities that the result is an enrichment of the associated transcripts equal to the enrichment of protein-coding transcripts. Moreover, we found lncRNAs in different ribosomal fractions, so the alternative explanation would require the involvement of multiple different protein complexes.

So why do lncRNAs associate with ribosomes? The possibility that these lncRNAs all code for proteins was recently eliminated by in-depth comparison of ribosome occupancy around translation termination codons [17]. lncRNAs did not show a steep drop in ribosomal binding after the translation termination codons (determined by the ribosome release score), as was seen for protein-coding genes. However, that does not exclude the possibility that ribosomes spuriously bind initiation codons in lncRNAs. In our data, the amount of ribosomes per lncRNA correlates with lncRNA length, maximum ORF length and the number of ORFs present per lncRNA, but those three factors are not independent of each other.

It is possible that one of the processes that keep lncRNAs at ribosomes is nonsense-mediated decay (NMD). NMD functions via ribosomal binding and has previously been described as a possible breakdown route of the noncoding RNA *GAS5*[35]. However, if NMD of a transcript results in such strong enrichment in the ribosomal fractions as observed in our experiments, it would mean that under standard culturing conditions a very significant portion of transcripts at ribosomes are engaged in NMD and not in active translation.

Arguably the most attractive hypothesis is that lncRNAs have functional roles in regulating translation. This could be a general phenomenon in which the lncRNAs occupy the ribosomes to keep them in a poised state and inhibit the energetically expensive process of translation until specific stimulatory cues are received. Alternatively, lncRNAs could regulate translation of specific protein-coding transcripts, for example by sequence-specific pairing. Indeed, recent data show that at least some lncRNAs associate with ribosomes to exert such a function [36]. For another class of noncoding RNAs, the microRNAs, similar roles have also been described [34]. One specific lncRNA, the antisense lncRNA of Uchl1, has been shown to regulate the association of sense Uchl1 with active polysomes in mice [36]. This regulatory function was partially established via the sequence homology between the lncRNA and the target mRNA. Translation regulatory mechanisms based on sequence homology have also been found for noncoding transcripts in bacteria [37]. Of the 25 antisense lncRNAs expressed in our data, only three pairs had both partners expressed and showed subcellular co-localization: *DYNLL1* and *DYNLL1-AS1*, *PCBP1* and *PCBP1-AS1*, and *WAC* and *WAC-AS1* (Additional file 10). The fact that we found so few co-localizing sense-antisense pairs makes it unlikely that a similar mechanism is abundant in the human system studied here.

## Conclusions

Our data show that different subcellular compartments differ significantly in RNA content, especially when the nucleus is compared to the ribosomal fractions. The lncRNAs expressed in this cell line are found in all subcellular samples and show an intricate correlation profile to protein-coding transcripts. Most lncRNAs are enriched in the cytosolic (free and the 40S/60S) samples and in the subcellular samples containing one, two or three ribosomes. The fact that lncRNAs show enrichment in diverse subcellular fractions and not only the nucleus suggests that lncRNAs may have a wider range of functions than currently anticipated. Our study provides insight into this diversity and our data can serve as a valuable resource for the functional characterization of individual lncRNAs.

## Materials and methods

### Accession numbers

All next-generation sequencing data used in this study can be downloaded from EMBL European Nucleotide Archive [PRJEB5049].

### Cell culture and media

Human colon cancer cells carrying a doxycycline-inducible short hairpin RNA against B-catenin (LS-174 T-pTER-*β*-catenin [23]) were cultured in 1X DMEM + GIBCO GlutaMAX™ (Life Technologies, Carlsbad, CA, USA) supplemented with 10% fetal calf serum and penicillin streptomycin. Cells were harvested during the exponential growth phase.

### Ribosome fractionation

All steps of the mono- and polyribosome profiling protocol were performed at 4°C or on ice. Gradients of 17% to 50% sucrose (11 mL) in gradient buffer (110 mM KAc, 20 mM MgAc and 10 mM HEPES pH 7.6) were poured the evening before use. Three replicates of 15 cm dishes with LS-174 T-pTER-*β*-catenin cells were lysed in polyribosome lysis buffer (110 mM KAc, 20 mM MgAc, 10 mM HEPES, pH 7.6, 100 mM KCl, 10 mM MgCl, 0.1% NP-40, freshly added 2 mM DTT and 40 U/mL RNasin (Promega, Madison, WI, USA)) with help of a Dounce tissue grinder (Wheaton Science Products, Millville, NJ, USA). Lysed samples were centrifuged at 1200 g for 10 min to remove debris and loaded onto the sucrose gradients. The gradients were ultra-centrifuged for 2 h at 120,565 g in an SW41 Ti rotor (Beckman Coulter, Indianapolis, IN, USA). The gradients were displaced into a UA6 absorbance reader (Teledyne ISCO, Lincoln, NE, USA) using a syringe pump (Brandel, Gaithersburg, MD, USA) containing 60% sucrose. Absorbance was recorded at an optical density of 254 nm. Fractions were collected using a Foxy Jr Fraction Collector (Teledyne ISCO). Corresponding fractions from each of the three replicates were merged prior to RNA isolation.

### Nuclei isolation

Pelleted nuclei of LS-174 T-pTER-*β*-catenin cells were obtained by centrifugation at 1200 g after whole-cell lysis prior to ribosome fractionation (see previous section). To exclude the presence of rough endoplasmic reticulum and thus validate the purity of the isolated nuclei, nuclear staining and imaging were performed (Additional file 1).

### RNA sequencing library preparation

Total RNA was isolated from purified nuclei using the TRIzol® reagent (#15596-026, Invitrogen, Life Technologies). RNA derived from triplicate mono- and polyribosome fractionation experiments was purified using TRIzol® LS reagent (#10296-028, Invitrogen, Life Technologies). Isolated RNA from the pooled triplicate fractions corresponded to the (A1 + 2) non-ribosome bound RNA, (B1) 40S subunit, (B2) 60S subunit, (C) 80S ribosome, (D) 2 ribosomes, (E) 3 ribosomes, (F) 4 ribosomes, (G) 5 ribosomes and (H) 6 ribosomes and (I) more than 6 ribosomes (Figure 1). For RNA-seq, RNA derived from A1 + 2 (non-ribosome bound RNA) and B1 + B2 (individual ribosomal subunits) was pooled prior to library preparation. RNA-seq libraries were prepared from rRNA-depleted RNA (Ribo-Zero™ Magnetic Gold Kit for Human/Mouse/Rat (MRZG12324, Epicentre®, Madison, WI, USA)) using the SOLiD™ Total RNA-seq kit (#4445374, Life Technologies). All libraries were sequenced on the SOLiD™ 5500 Wildfire system (40 bp fragment reads).

### Data analysis

RNA-seq reads were mapped using Burrows-Wheeler Aligner [38] (BWA-0.5.9) (settings: -c -l 25 -k 2 -n 10) onto the human reference genome hg19. Only uniquely mapped, non-duplicate reads were considered for further analyses. Reads that mapped to exons were used to determine the total read counts per gene. Exon positions were based on the GENCODE v18 annotation [24]. The polyribosomal samples (from two to seven or more associated ribosomes) yielded 13 to 32 million reads. For the non-polyribosomal samples (nuclear, free cytosolic, combined 40S and 60S, and 80S (monosomes)), data from three sequencing lanes (technical replicates) were merged yielding 6 to 64 million reads. Data analysis was performed on the genes with GENCODE gene_type: protein coding, antisense, processed transcript, long intergenic noncoding RNA and snRNA/snoRNAs. Filtering was performed on the read count per gene over all samples combined. The per transcript sum of the sequencing reads in all samples showed a bimodal distribution (Additional file 11). Based on these data we used a total read count threshold of 2,500 per transcript to select the expressed genes. Genes with total read count below 2,500 were filtered out, leaving 7,734 genes for further analysis. Subsequently, normalization was performed using the DEseq [39] to correct for library size and technical biases. Gene clustering was performed using a model-based clustering approach with the R package HTSCluster [25]. The protein coding-lncRNA correlation matrix (Figure 3) was calculated using Spearman rank correlation. The matrix was visualized after hierarchical clustering using Euclidean distance with complete linkage. Median transcript length and coding sequence length were calculated for the protein-coding genes using annotation from Ensembl. The maximum lncRNA ORFs were predicted using a custom Perl script aimed at finding reading frames with in-frame START and STOP codons, without intervening in-frame STOP codons.

### Quantitative PCR analysis

Quantitative PCR analysis was performed on cDNA derived from total RNA of cytosolic, nuclear and pooled polyribosomal RNA. The RT reaction was performed on 1 μg of total RNA using oligo d(T) primers and the high capacity cDNA reverse transcription kit (Life Technologies, #4368814). Three primer sets were designed per lncRNA. Quantitative PCR reactions were performed in 20 μl reactions using 2 ng of cDNA and iQ™ SYBR® Green Supermix (Bio-Rad, Hercules, CA, USA, #170-8880) on a MyIQ2 Real-time PCR detection system (Bio-Rad).

## Abbreviations

bp: Base pairs; CPM: Counts per million; lncRNA: Long noncoding RNA; NMD: Nonsense mediated decay; ORF: Open reading frame; PCR: Polymerase chain reaction; RNA-seq: RNA-sequencing; rRNA: Ribosomal RNA; RT: Reverse transcription; sncRNA: Small noncoding RNA; snoRNA: Small nucleolar RNA; snRNA: Small nuclear RNA.

## Competing interests

The authors declare that they have no competing interests.

## Authors’ contributions

SvH, MvI, EC and MS wrote the manuscript. MvI, MS, SB and JJ performed RNA-seq data analyses. SvH performed cell culture, nuclei isolation and RNA-seq experiments. SvH and PBE performed polysomal fractionation experiments. WH and EdB performed next-generation sequencing. SvH, EC, MS and AWM designed the experiments. All authors contributed to scientific discussions, and read and approved the final version of the manuscript.

## Supplementary Material

Additional file 1 Microscopy images of purified nuclei. Click here for file

Additional file 2 Table showing the raw read counts of all sequenced fractions per gene. Click here for file

Additional file 3 **Scatter plot illustrating the contribution of sncRNAs, protein-coding transcripts and lncRNAs to the observed correlation between the nuclear and >6 ribosome sample (in relation to Figure** 2**A).** Click here for file

Additional file 4 **Bar plot depicting the contents of each sequenced sample in reads per kilobase per million instead of CPMs (in relation to Figure** 2**B).** Click here for file

Additional file 5 **Table showing the normalized read counts for each cluster in Figure** 4**A.** Click here for file

Additional file 6 Four heatmaps illustrating the effects on k-means clustering when 9 to 12 clusters are required. Click here for file

Additional file 7 Effects on clustering and transcript localization when all sncRNAs are removed from the data. Click here for file

Additional file 8 Distribution of lncRNAs over the polyribosomal fractions in relation to the number of ORFs detected per transcript. Click here for file

Additional file 9 Figure showing the enrichment of lncRNAs in the nucleus or cytosol by qPCR analysis. Click here for file

Additional file 10 **Table with the results of the antisense lncRNA versus senseprotein-codingtranscript co-localization analysis.** Click here for file

Additional file 11 Graph showing the bimodal distribution of sequencing reads over all sequencing data. Click here for file
