# Supplementary material for: Extensive localization of long noncoding RNAs to the cytosol and mono- and polyribosomal complexes
Source: Genome Biol. 2014 Jan 7;15(1):R6. doi: 10.1186/gb-2014-15-1-r6 (PMC4053777; doi:10.1186/gb-2014-15-1-r6)
Supplement: Additional file 1 — Microscopy images of purified nuclei. [file gb-2014-15-1-r6-S1.pdf]

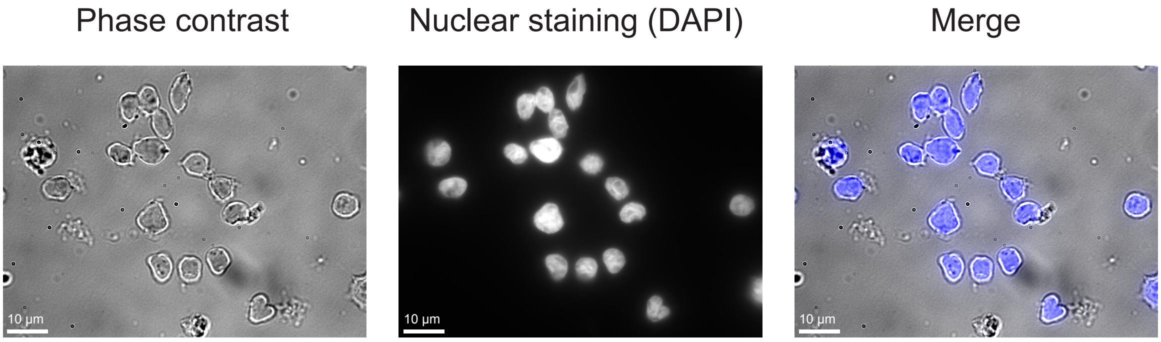

Additional file 1) Quality controls for the purity of nuclear isolation. Following cell lysis, nuclei were pelleted at 1200g and used for imaging and RNA isolation. Phase contrast microscopy, DAPI staining and merge of the two showing the puritiy of the nuclei isolation.
