# Supplementary material for: Extensive localization of long noncoding RNAs to the cytosol and mono- and polyribosomal complexes
Source: Genome Biol. 2014 Jan 7;15(1):R6. doi: 10.1186/gb-2014-15-1-r6 (PMC4053777; doi:10.1186/gb-2014-15-1-r6)
Supplement: Additional file 3 — Scatter plot illustrating the contribution of sncRNAs, protein-coding transcripts and lncRNAs to the observed correlation between the nuclear and >6 ribosome sample (in relation to Figure 2A). [file gb-2014-15-1-r6-S3.pdf]

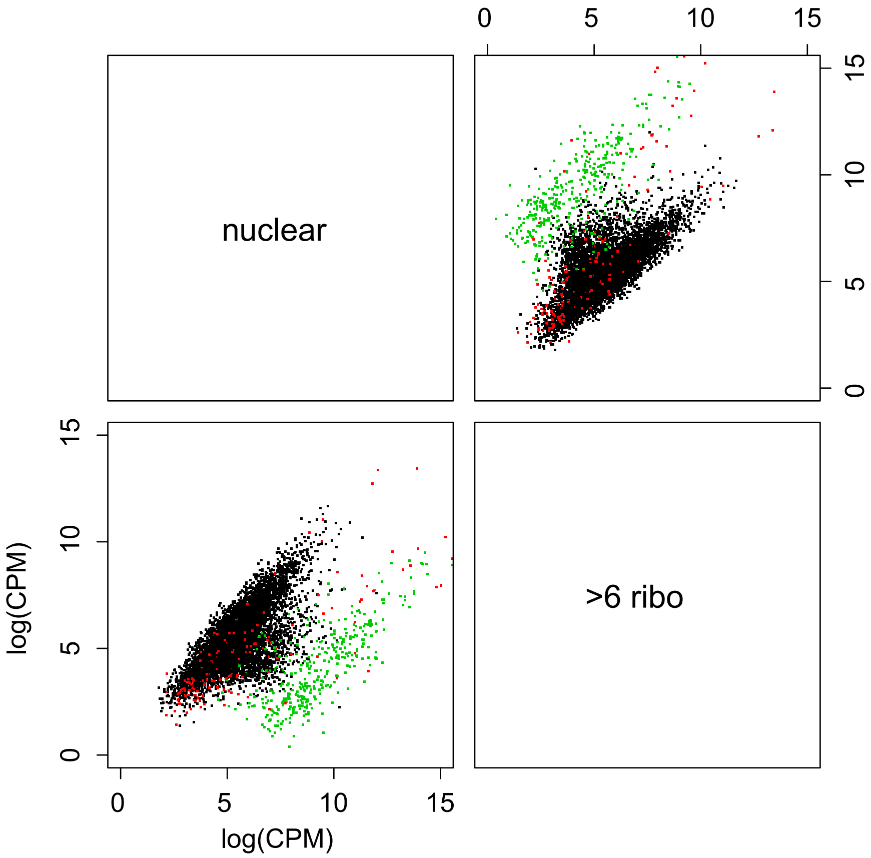


**Additional file 3) Correlation scatters of the nuclear fraction versus the >6 ribosomes fraction.** The different clouds in this scatterplot represent the protein coding transcripts (black), the small noncoding RNAs (green) and the lncRNAs (red).
