# Supplementary material for: Extensive localization of long noncoding RNAs to the cytosol and mono- and polyribosomal complexes
Source: Genome Biol. 2014 Jan 7;15(1):R6. doi: 10.1186/gb-2014-15-1-r6 (PMC4053777; doi:10.1186/gb-2014-15-1-r6)
Supplement: Additional file 4 — Bar plot depicting the contents of each sequenced sample in reads per kilobase per million instead of CPMs (in relation to Figure 2B). [file gb-2014-15-1-r6-S4.pdf]

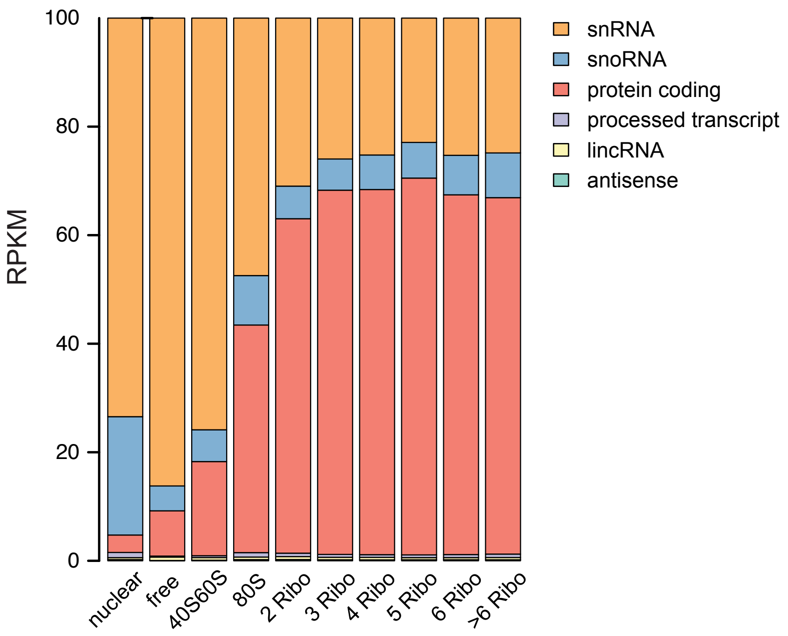

Additional file 4) Reads per kilobase per million (RPKM) normalized content of each sequenced fraction. Types of transcripts (both coding and noncoding) are indicated with different colors.
