# Supplementary material for: Extensive localization of long noncoding RNAs to the cytosol and mono- and polyribosomal complexes
Source: Genome Biol. 2014 Jan 7;15(1):R6. doi: 10.1186/gb-2014-15-1-r6 (PMC4053777; doi:10.1186/gb-2014-15-1-r6)
Supplement: Additional file 6 — Four heatmaps illustrating the effects on k-means clustering when 9 to 12 clusters are required. [file gb-2014-15-1-r6-S6.pdf]

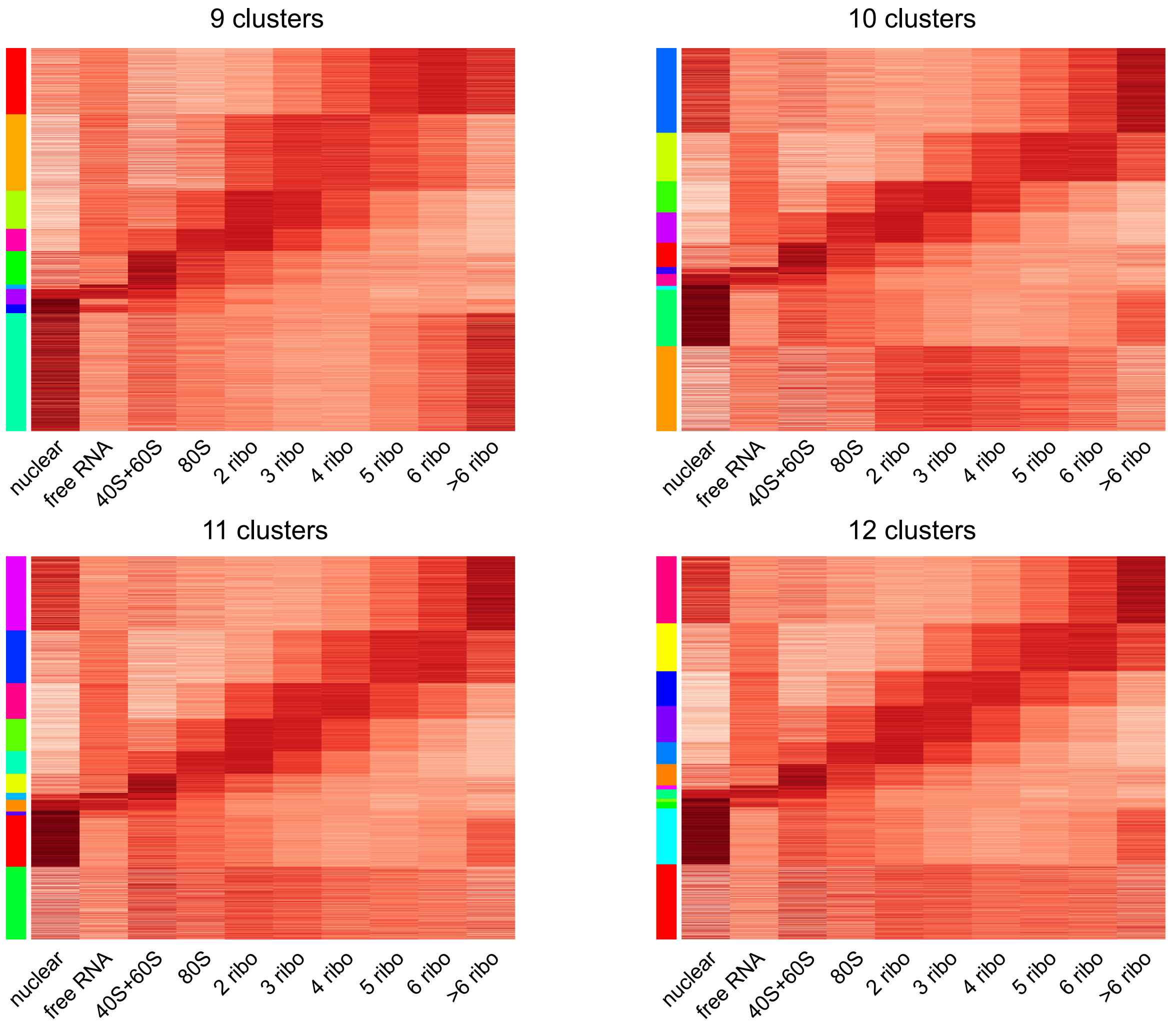

Additional file 6) k-means clustering results do not depend on the number of clusters used. Results for clustering over the different fractions are shown for 9, 10, 11 and 12 clusters.
