# Supplementary material for: Extensive localization of long noncoding RNAs to the cytosol and mono- and polyribosomal complexes
Source: Genome Biol. 2014 Jan 7;15(1):R6. doi: 10.1186/gb-2014-15-1-r6 (PMC4053777; doi:10.1186/gb-2014-15-1-r6)
Supplement: Additional file 7 — Effects on clustering and transcript localization when all sncRNAs are removed from the data. [file gb-2014-15-1-r6-S7.pdf]

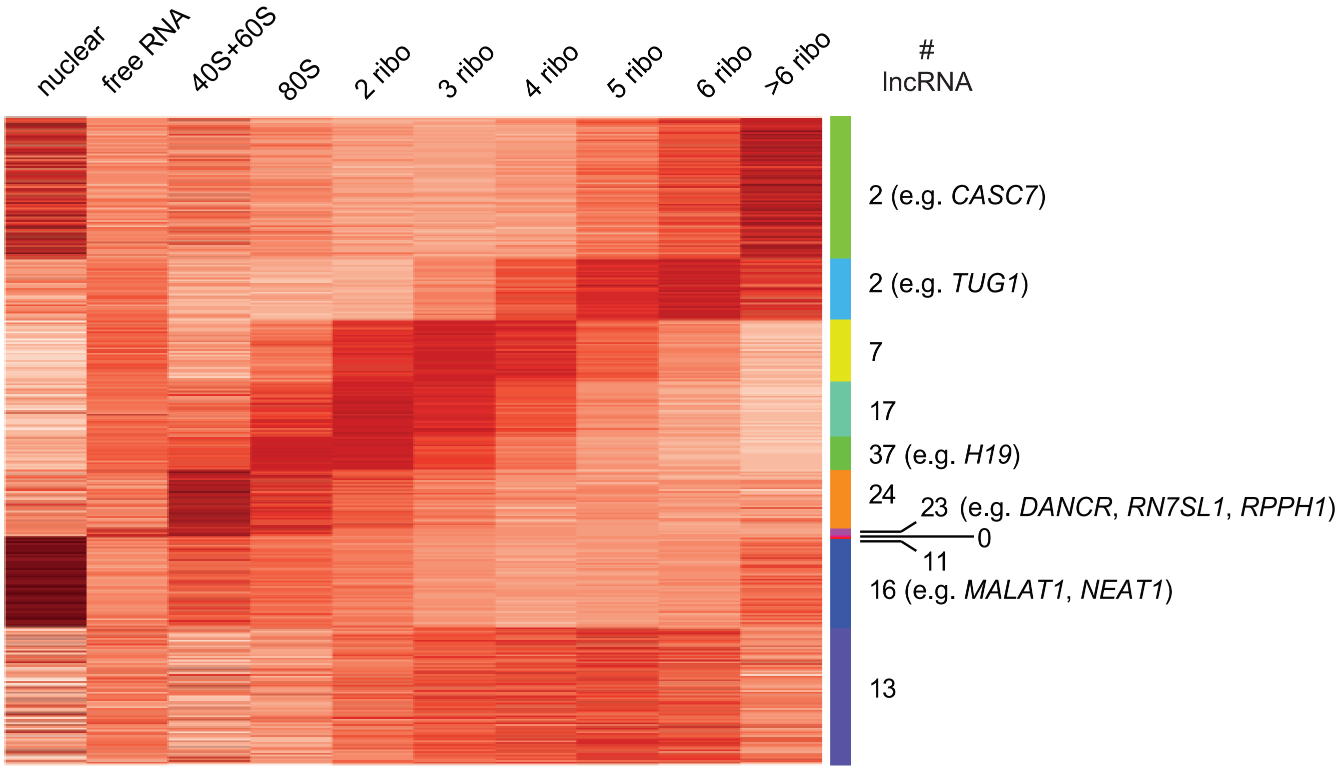

Additional file 7) Clustering of the data without sncRNAs does not affect the finding of lncRNAs in cytosolic and ribosomal fractions. Read counts of only the protein-coding transcripts and lncRNAs were normalized across the samples and k-means clustering was applied generating 11 clusters. The number of lncRNAs present in each of the clusters is indicated on the right.
