# Supplementary material for: Extensive localization of long noncoding RNAs to the cytosol and mono- and polyribosomal complexes
Source: Genome Biol. 2014 Jan 7;15(1):R6. doi: 10.1186/gb-2014-15-1-r6 (PMC4053777; doi:10.1186/gb-2014-15-1-r6)
Supplement: Additional file 8 — Distribution of lncRNAs over the polyribosomal fractions in relation to the number of ORFs detected per transcript. [file gb-2014-15-1-r6-S8.pdf]

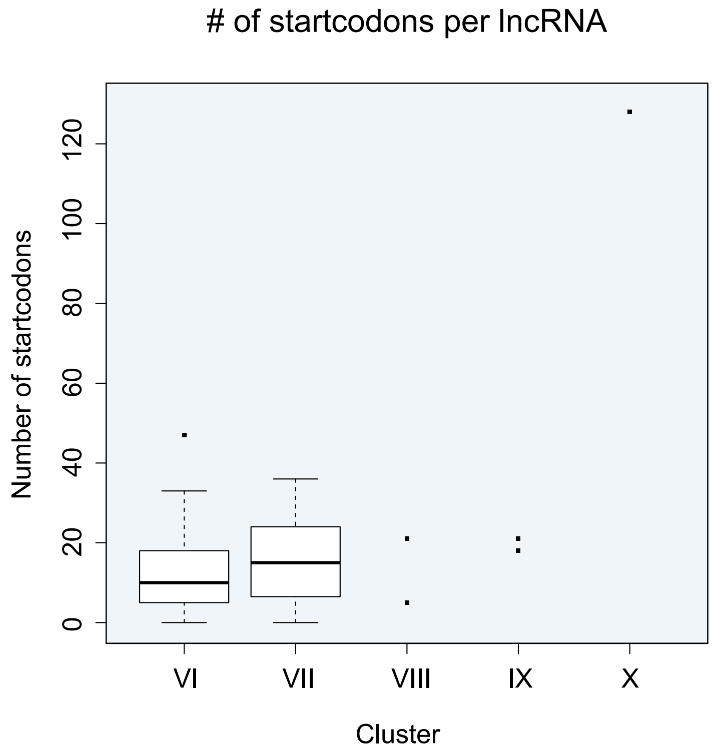

Additional file 8) Number of startcodons per lncRNA per cluster. For 5 ribosomal clusters, the number of potential ORFs is determined by counting the ATG translational start sites per transcript.
