# Supplementary material for: Extensive localization of long noncoding RNAs to the cytosol and mono- and polyribosomal complexes
Source: Genome Biol. 2014 Jan 7;15(1):R6. doi: 10.1186/gb-2014-15-1-r6 (PMC4053777; doi:10.1186/gb-2014-15-1-r6)
Supplement: Additional file 9 — Figure showing the enrichment of lncRNAs in the nucleus or cytosol by qPCR analysis. [file gb-2014-15-1-r6-S9.pdf]

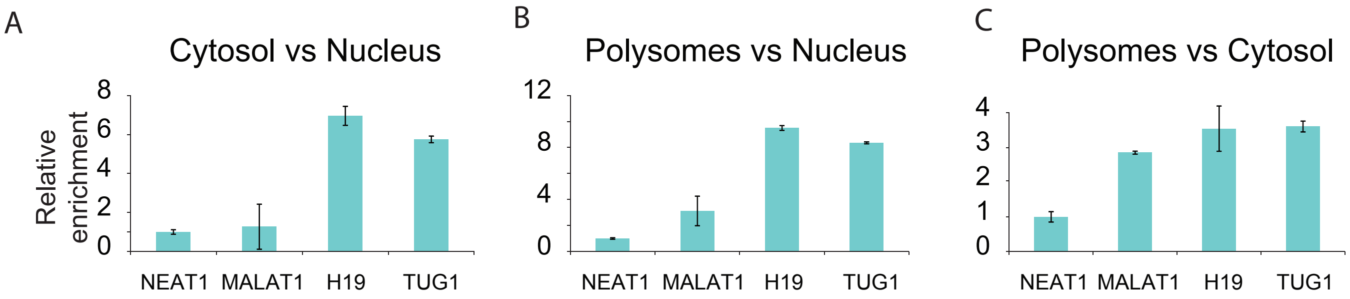

Additional file 9) Subcellular enrichment of specific lncRNAs is confirmed by qPCR. Relative enrichment of the four tested lncRNAs is represented as delta(deltaCT). CT values were normalized first per sample to H19, resulting in a deltaCT value. Relative differences between lncRNAs, between subcellular samples are determined by calculating the difference between the deltaCTs between samples, resulting in delta(deltaCT) values. delta(deltsCT) values were determined between A) cytosol and nucleus, B) polysomes and nucleus and C) polysomes and cytosol. The lowest delta(deltaCT) is set to 1 (NEAT1 in all panels). Error bars depict the standard deviation of three different primersets used per lncRNA.
