# Supplementary material for: Extensive localization of long noncoding RNAs to the cytosol and mono- and polyribosomal complexes
Source: Genome Biol. 2014 Jan 7;15(1):R6. doi: 10.1186/gb-2014-15-1-r6 (PMC4053777; doi:10.1186/gb-2014-15-1-r6)
Supplement: Additional file 11 — Graph showing the bimodal distribution of sequencing reads over all sequencing data. [file gb-2014-15-1-r6-S11.pdf]

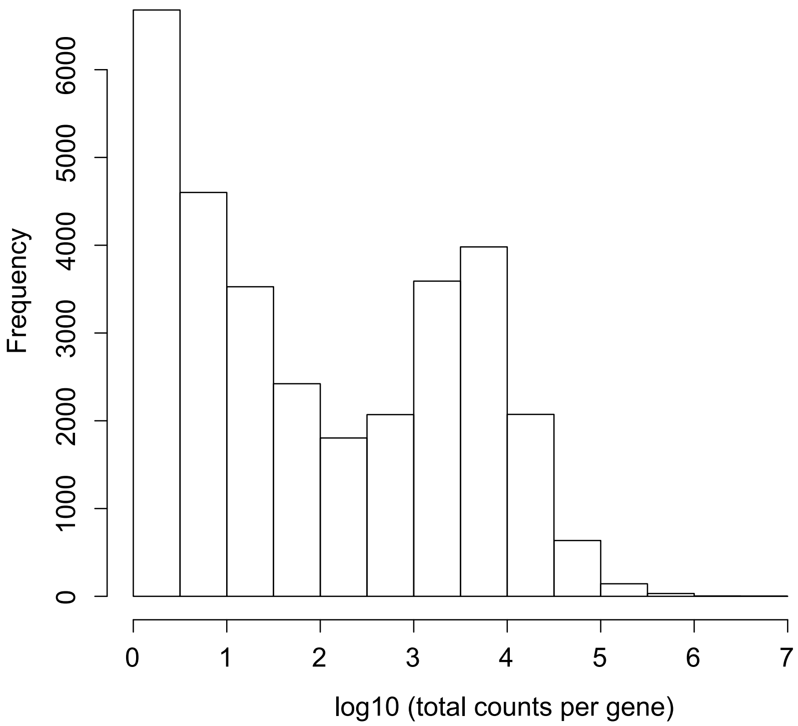

Additional file 11) A bimodal distribution in total counts per gene can be observed in the sequencing data. Read counts for each transcript in all samples was summed and the distribution was determined.
